# Pretransplant Splenic Irradiation in Patients With Myeloproliferative Neoplasms

**DOI:** 10.1016/j.adro.2022.100964

**Published:** 2022-04-10

**Authors:** Sara Beltrán Ponce, Saurabh Chhabra, Parameswaran Hari, Selim Firat

**Affiliations:** a Department of Radiation Oncology, Medical College of Wisconsin, Milwaukee, Wisconsin; b Division of Hematology/Oncology, Department of Medicine, Medical College of Wisconsin, Milwaukee, Wisconsin; c Blood and Marrow Transplantation & Cellular Therapy Program, Froedtert & Medical College of Wisconsin, Milwaukee, Wisconsin

## Abstract

**Purpose:**

Allogeneic hematopoietic cell transplantation (HCT) serves as the only curative treatment option for patients with myelofibrosis and other myeloproliferative neoplasms. Splenomegaly commonly manifests in patients with myeloproliferative neoplasms and can lead to delayed or poor engraftment, increased transfusion burden, and worse survival. Methods to decrease the effect of splenomegaly include splenectomy and splenic irradiation. We sought to report on clinical outcomes for patients treated with splenic irradiation as part of their transplant conditioning.

**Methods and Materials:**

Patients with splenomegaly measuring greater than 22 cm were referred for splenic irradiation. They received radiation to the entire spleen to 10 Gy in 5 fractions using 3-dimensional conformal radiation with anteroposterior/posteroanterior or opposed tangent fields. Blood counts were monitored closely on treatment. Changes in splenic size were measured using first and last treatment image guided radiation therapy and pre- and posttransplant diagnostic imaging.

**Results:**

Seventeen patients completed pretransplant splenic irradiation between 2012 and 2021. Median platelet, white blood cell, and hemoglobin levels decreased on treatment. One patient required platelet transfusion and 3 required packed red blood cell transfusions. Mean decrease in spleen size during radiation was -8.5% in the craniocaudal dimension. Prolonged decreases, measured 2 to 12 months after transplant, averaged 14.64%. All patients engrafted. Fourteen (82.4%) were alive at time of analysis with median follow-up of 4.2 years from hematopoietic cell transplantation.

**Conclusions:**

Splenic irradiation offers a safe method of managing significant splenomegaly as part of transplant conditioning. Transplant outcomes in this series were excellent. Prospective data may be beneficial to determine the absolute benefit of this addition to pretransplant conditioning in this patient population.

## Introduction

For patients with myelofibrosis and other myeloproliferative neoplasms (MPNs), allogeneic hematopoietic cell transplantation (HCT) serves as the only potentially curative treatment option.^1^^,^^2^ Splenomegaly is a common clinical manifestation of MPNs and is a result of increased splenic hematopoiesis to counter decreased functionality of bone marrow in these patients.^3^ Before HCT, splenomegaly is usually managed with drug treatment, including hydroxyurea and ruxolitinib, radiation, or, in severe cases, splenectomy.^4^, ^5^, ^6^ These management strategies serve to both improve cytopenias caused by splenic sequestration and alleviate symptomatic concerns such as abdominal pain, nausea, early satiety, and distention. However, these measures are often ineffective and leave patients with continued symptoms and the risk of losing the only site of hematopoiesis when the marrow fibrosis is severe.^7^ In the context of allogeneic transplant, splenomegaly can lead to delayed or poor engraftment, high transfusion burden, and worsened survival.

As conditioning for curative intent transplantation begins, treatment goals shift to engraftment, prevention of graft failure and disease relapse, and improved survival. There is evidence to suggest that splenomegaly is associated with decreased rates of engraftment and overall survival.^8^, ^9^, ^10^ There are several options used to manage this in the pretransplant setting, including surgical resection and splenic irradiation. Splenectomy has been associated with improved transplant outcomes and faster posttransplant hematopoietic recovery, but it is also associated with increased morbidity.^8^^,^^11^^,^^12^ Low-dose splenic irradiation has been safely used in pretransplant conditioning in chronic myeloid leukemia with a potential relapse reduction in certain subgroups.^13^^,^^14^

There are limited data regarding the potential use of pretransplant radiation for patients with myelofibrosis. A single-center retrospective review (n = 44) comparing outcomes in those who completed pretransplant splenic irradiation (PrTSI) (n = 11) and nonirradiated patients (n = 33) receiving allogeneic transplant for myelofibrosis (30 primary and 14 secondary) noted an improvement in 2-year overall survival from 48% in nonirradiated patients to 72% in irradiated patients, though statistical significance was not evident in this limited data set.^15^ Two small series of 2 and 8 patients each noted that PrTSI is safe in the myelofibrosis population and may allow patients’ transplant kinetics to mirror that of the nonsplenomegaly myelofibrosis population.^16^^,^^17^

Since 2012, patients with MPN and clinically significant splenomegaly (>22 cm in the craniocaudal dimension) undergoing HCT at the Medical College of Wisconsin have received splenic irradiation immediately before starting the conditioning regimen with the goals of reducing splenic size and reducing the likelihood of posttransplant splenic sequestration. In the following sections, we report on the treatment technique, treatment side effects, and long-term outcomes for these patients.

## Methods and Materials

### Treatment planning

Patients were treated in the supine position with no contrast or fasting before simulation. Motion management was not used. Spleen was contoured as the gross tumor volume and treatment fields were shaped to cover the spleen tightly but excluded the left kidney and heart as much as possible. Dose to both kidneys and heart were tracked.

Most treatments were completed using 3-dimensional conformal radiation with anteroposterior, posteroanterior, or opposed tangential fields. However, normal tissue constraints for 2 cases required formulation of upper and lower spleen fields with a single isocenter to accommodate spleen size while respecting normal tissues.

### Radiation delivery

Patients were treated to a total of 10 Gy in 5 fractions at 2 Gy per fraction. Treatment started 5 business days before scheduled admission and was completed the day before admission. Daily  image guided radiation therapy (IGRT) was used.

Complete blood count (CBC) was monitored during the course of treatment to assess the need for transfusion. No treatment breaks were allowed for cytopenias.

### Transplant conditioning

Please refer to a previous publication for additional information on the conditioning regimens used in these patients and the transplant outcomes.^18^ As part of the protocol, patients with MPN with splenomegaly measuring greater than 22 cm were referred for splenic irradiation, with the final fraction completed 1 day before scheduled admission for conditioning.

### Data collection

All patients who underwent PrTSI between 2012 and 2021 were included in the analysis. Pretransplant CBC was available for all patients within 1 month of radiation treatment, with the majority of patients having a CBC within 1 week. Posttreatment CBC was obtained on the day after radiation treatment completion for all patients. Transfusion utilization during this period was also available.

Spleen size was measured craniocaudally using IGRT on the first and last day of radiation treatment to assess acute changes in splenic size from radiation. Long-term splenic size changes were measured using pretransplant computed tomography (CT) or ultrasound and posttransplant CT within 12 months. Engraftment was documented if neutrophil count was >500/mcL for 3 consecutive days.

This study was conducted in accordance with the Declaration of Helsinki. It was approved by the institutional ethics board of the Medical College of Wisconsin and a waiver of informed consent was granted based on minimal risk and the retrospective nature of the study.

## Results

Seventeen patients completed PrTSI between 2012 and 2021, including 4 females and 13 males (Table 1). Fourteen patients had a diagnosis of myelofibrosis, 1 had myelodysplastic syndrome/MPN, and 2 had an MPN not otherwise specified. The median patient age was 63 years (range, 46-74 years). Five patients (29.4%) also completed a single fraction of total body irradiation to 200 cGy, given as part of the conditioning regimen. Sixteen patients were on JAK inhibitors before transplant. Before treatment initiation, 4 patients noted fatigue, 2 patients noted early satiety, and 1 patient noted abdominal fullness. Postradiation symptom changes were not assessed as patients underwent transplant directly after the completion of PrTSI.

**Table 1:** Patient characteristics and outcomes

Characteristics	Outcomes
Patient characteristics	
Age (median, range)	63 (46-74)
Male (%)	13 (76%)
Spleen size reduction	
Treatment reduction (median, range)	7.3% (0%-22.5%)
Long-term reduction (median, range)	14.3% (-5.3%-36.5%)
Hematologic toxicities	
Change in platelet count (/µL) (median, range)	-19,000 (-108,000-31,000)
Change in leukocyte count (/µL) (median, range)	-6350 (-27,900-1600)
Change in hemoglobin (g/dL) (median, range)	-0.4 (-1.9-0.9)
Transplant outcomes	
Engraftment rate	100%
Median survival	4 years
Survival at time of review	82.4%

### Treatment toxicities

Median pretreatment platelet count was 109,000/µL (range, 14,000-531,000/µL). Median decrease during treatment was 19,000/µL (range, -31,000-108,000/µL). One patient required platelet transfusion during treatment. This patient had a pretreatment platelet count of 14,000/µL, an on-treatment nadir of 7000/µL, and a posttreatment platelet count of 14,000/µL, with 3 platelet transfusions occurring during the 7-day treatment window. The most notable change to blood counts was lymphocytes with a pretreatment median of 8000/µL (range, 0-41, 100/µL) and a median drop of 6350/µL (range, 1600-27, 900/µL), for a final median value of 2700/µL. Median pretreatment hemoglobin (Hgb) was 8.6 g/dL (range, 5.8-12.9 g/dL) with a mean drop of 0.4 g/dL (range, 0.9-1.9 g/dL). One patient had 3 packed red blood cell (pRBC) transfusions, and 2 patients had 1 pRBC transfusion each. One patient requiring pRBC transfusion had a pretreatment Hgb of 8.3 g/dL and an on-treatment nadir of 7.4 g/dL. A second patient had a pretreatment Hgb of 5.7 g/dL and a nadir of 5.5 g/dL, and the third had a pretreatment Hgb of 7.5 g/dL (nadir), was transfused on day 1, and remained above 8 g/dL throughout the remainder of treatment.

In addition to hematologic toxicities, 1 patient had grade 1 nausea, 1 patient had grade 1 fatigue, and 2 patients had grade 2 vomiting according to Common Terminology Criteria for Adverse Events version 5. There were no grade 3 or greater nonhematologic toxicities.

### Splenic size changes

Radiation treatment-related splenic size decreased in 14 patients and remained stable in 3. For all patients, the median change in splenic size with radiation was -7.3% in the craniocaudal dimension. Among those who had a size reduction, the median reduction was 8.0%, with a range from 3.9% to 22.5% (Table 2).

**Table 2:** Outcomes by patient including splenic size changes, radiation-related cytopenias, and survival

ID	Age	Sex	Spleen size change during radiation	Spleen size change in first year post-HCT	Radiation-related platelet change (/µL)	Radiation-related leukocyte change (/µL)	Radiation-related hemoglobin reduction (g/dL)	Total body irradiation	Engraftment		Survival		Survival length (years)		Neutrophil engraftment (days)	Platelet engraftment (days)
1	71	M	-8.3%	N/A	-8000	-6200	-0.9	N		Y		Y		4.3	16	112
2	54	F	-17.6%	-14.2%	+31,000	-1000	+0.1	N		Y		Y		0.4	15	16
3	60	M	-3.9%	N/A	-36,000	-7000	-0.5	N		Y		Y		4.9	18	30
4	74	M	-7.3%	-23.9%	+10,000	-2100	+0.2	N		Y		Y		4.2	17	131
5	67	F	-10.3%	-14.4%	-103,000	-16,500	-0.4	N		Y		N		0.7	23	-
6	57	M	-7.3%	-6.8%	-36,000	-1500	-0.4	N		Y		Y		3.0	17	20
7	70	M	-22.5%	N/A	-15,000	-10,000	-0.1	N		Y		Y		5.7	16	20
8	68	M	0%	-15.3%	-72,000	-7800	-0.9	Y		Y		Y		8.9	25	108
9	63	M	-16.7%	-36.5%	+8000	-11,800	-1.8	N		Y		N		0.3	24	-
10	46	M	0%	N/A	-74,000	+1600	+ 0.6	Y		Y		Y		4.0	16	32
11	53	F	-5.9%	-12%	-76,000	-1000	-1.3	N		Y		N		0.6	24	-
12	48	M	0%	-2.3%	0	0	+ 1	Y		Y		Y		0.5	18	29
13	46	F	-6%	0%	-19,000	-6300	-1.9	N		Y		Y		4.6	22	91
14	61	M	-7.7%	-18.9%	-9000	-29,000	+ 0.3	Y		Y		Y		1.1	21	27
15	65	M	-15%	-16.7%	-34,000	-6400	+ 0.2	N		Y		Y		4.3	32	107
16	72	M	-5.7%	5.3%	-108,000	-27,900	-1.7	N		Y		Y		5.7	17	26
17	66	M	-9.6%	N/A	-3000	-900	+ 0.9	Y		Y		Y		0.3	26	48

*Abbreviation:* F = female; HCT = hematopoietic cell transplantation; M = male; N = no; N/A = not applicable; Y = yes.

Preradiation and posttransplant imaging for splenic size comparison were available for 12 patients. The median decrease in splenic size between preradiation and posttransplant images, with imaging obtained at a mean of 4.8 months posttransplant (range, 2-12 months), was 14.30% (range, -5.3%-36.5%). One patient had stable spleen size and 1 had an increase in size of 5.3%. This patient did not relapse in this time frame, and etiology of the increased splenic size is unknown. Among those who had a prolonged decrease in spleen size, the median decrease was 14.85% (range 2.3%-36.50%).

### Transplant outcomes

All 17 patients achieved engraftment. The median time to neutrophil engraftment was 18 days (range, 15-32 days), and median time to platelet engraftment was 31 days (range, 26-131). Three patients did not engraft platelets while all engrafted neutrophils. Fourteen patients (82.4%) were alive at the time of analysis. These patients have a median follow-up of 4.2 years from HCT (range, 0.3-8.9 years). Day 100 all-cause mortality was 0%. Three patients died at last follow-up, 1 from sepsis, 1 from graft versus host disease, and 1 from acute myeloid leukemia. These patients correlate with the 3 who did not achieve platelet engraftment and have a median survival of 218 days from transplant (range, 118-244 days). Median survival was 4.0 years from HCT for the entire cohort (range, 0.3-8.9 years).

### Discussion

Most of the previous experience with splenic irradiation in patients with MPN involves its use for symptom palliation. Although splenic irradiation in this protocol is an adjunct to standard HCT conditioning, which can decrease spleen size in isolation, there was still a notable decrease in spleen size, with a median decrease of 7.3% during treatment and a continued, posttransplant decrease of 14.3%. This has been similarly commented on in literature specific to palliation, but it is important to note for these patients as well.^7^^,^^19^ In addition, the potential for symptom palliation in this setting is unknown as it is impossible to assess the direct effect of radiation in this cohort due to transplantation directly after completion of treatment.

In addition to splenectomy, which suggests morbidity and mortality rates as high as 30% and 8%, respectively, splenic irradiation offers a safe, alternative option for reducing and/or controlling spleen size to promote engraftment.^20^^,^^21^ In this series, there was limited toxicity and were no deaths within 3 months after completion of splenic irradiation. Splenic irradiation may be considered a safer, better tolerated alternative to splenectomy in patients with myelofibrosis and MPNs who require some form of splenic management immediately before and in conjunction with transplant conditioning for improved transplant outcomes, including hematopoietic recovery.

Most patients with MPN are on JAK inhibitor before transplant, and these agents, when discontinued immediately pretransplant, can lead to rapid rebound splenomegaly.^22^^,^^23^ We did not observe this in any of our 16 patients who tapered off JAK inhibitor for allogeneic transplant. In the 1 patient with a small increase in spleen size 4 months after transplant, the splenic size still remained smaller than the initial preradiation and transplant dimension.

Transplant outcomes in this review demonstrated engraftment in all 17 patients. Published data on engraftment suggest that 2% to 24% of patients with MPN and splenomegaly fail to engraft.^24^ Our data suggest that splenic irradiation may have a role in improved engraftment rates.

Fourteen of 17 patients were alive at the time of analysis (82.4%); these survival data also favor the use of splenic irradiation in patients with MPN with splenomegaly. In another series that compared HCT with and without splenic irradiation, adding splenic irradiation to pretransplant conditioning led to a trend toward improved survival at 2 years, with 72% of irradiated patients and 48% of nonirradiated patients surviving.^15^ The lack of significance may be due to the small sample size and short length of follow-up.

The limitations of this study are its retrospective nature, small sample size, and use of single-center data set. In addition, the available splenic imaging to measure the treatment size change comprised of daily IGRT images completed for treatment set-up, which have decreased image quality compared with diagnostic CT scans. Long-term size changes used both CT and ultrasound imaging, with 9 patients using only CT, 1 using only ultrasound, and 1 using a combination. The 2 patients with varying pre- and posttreatment imaging modalities may have inconsistency in their size assessment; however, no additional imaging was available at the time of publication. Lastly, this study did not assess changes in patient-reported outcomes for symptoms such as bloating, abdominal pain, nausea, and early satiety, which were not collected. These data would allow determination as to whether this protocol can serve a dual purpose of transplant conditioning and palliation for these patients. It is possible that improving these symptoms may allow patients to have improved nutrition and oral intake due to lessened nausea and early satiety.

In conclusion, PrTSI for patients with myelofibrosis and MPNs with significant splenomegaly is safe and offers a low side effect profile with excellent transplant outcomes. Prospective data may be beneficial to elucidate the absolute benefit of this aspect of pretransplant conditioning as well as a potential dual benefit of symptom palliation.
